# Supplementary material for: PHLPP2 is a pseudophosphatase that lost activity in the metazoan ancestor
Source: Proc Natl Acad Sci U S A. 2025 Apr 1;122(14):e2417218122. doi: 10.1073/pnas.2417218122 (PMC12002173; doi:10.1073/pnas.2417218122)
Supplement: Supplementary file 1 — Appendix 01 (PDF) [file pnas.2417218122.sapp.pdf]

Supporting Information

PHLPP2 is a pseudophosphatase that lost activity in the metazoan ancestor

Tarik Husremović[a,1], Vanessa Meier[a,1], Lucas Piëch[a,b], Katharina M. Siess[a], Sumire Antonioli[a,b], Irina Grishkovskaya[c], Nikoleta Kircheva[d], Silvia E. Angelova[d,e], Karoline Wenzl[a], Andreas Brandstätter[f], Jiri Veis[a], Fran Miočić-Stošić[a,b,g], Dorothea Anrather[h,i], Markus Hartl[h,i], Linda Truebestein[a], Luis M. Cerron-Alvan[a,b,j], Martin Leeb[a,j], Bojan Žagrović[a,g], Stephan Hann[f], Christoph Bock[k,l], Egon Ogris[a], Todor Dudev[m], Nicholas A.T. Irwin[n], David Haselbach[c] and Thomas A. Leonard[a,2]

[a]Max Perutz Labs, University of Vienna and Medical University of Vienna, Vienna 1030, Austria

[b]Vienna BioCenter PhD Program, a Doctoral School of the University of Vienna and the Medical University of Vienna, Vienna 1030, Austria

[c]Research Institute of Molecular Pathology, Vienna BioCenter, Vienna 1030, Austria

[d]Institute of Optical Materials and Technologies "Acad. J. Malinowski", Bulgarian Academy of Sciences, Sofia 1113, Bulgaria

[e]University of Chemical Technology and Metallurgy, Sofia 1756, Bulgaria

[f]Department of Chemistry, Institute of Analytical Chemistry, University of Natural Resources and Life Sciences, Vienna 1190, Austria

[g]Department of Structural and Computational Biology, Center for Molecular Biology, University of Vienna, Vienna 1030, Austria

[h]Max Perutz Labs, Mass Spectrometry Facility, Vienna Biocenter Campus, Vienna 1030, Austria

[i]Department of Biochemistry and Cell Biology, Center for Molecular Biology, University of Vienna, Vienna 1030, Austria

[j]Department of Microbiology, Immunobiology and Genetics, Center for Molecular Biology, University of Vienna, Vienna 1030, Austria

[k]Research Center for Molecular Medicine, Austrian Academy of Sciences, Vienna 1090, Austria

[l]Center for Medical Data Science, Institute of Artificial Intelligence, Medical University of Vienna, Vienna 1090, Austria

[m]Faculty of Chemistry and Pharmacy, Sofia University "St. Kliment Ohridski", Sofia 1164, Bulgaria

[n]Gregor Mendel Institute, Austrian Academy of Sciences, Vienna BioCenter, Vienna 1030, Austria

[1]T.H. and V.M. contributed equally to this work

[2]Correspondence: thomas.leonard@meduniwien.ac.at

Contains:

Supplementary Methods
Supplementary Figures 1-9
Supplementary Table 1
Supplementary References

Supplementary Methods

Protein expression and purification

Recombinant human PHLPP2 and mutants thereof were expressed as N-terminal GST fusion proteins in baculovirus-infected *Spodoptera frugiperda* (Sf9) cells using a pFastBac Dual vector. Cells were harvested and lysed in buffer containing 50 mM Tris, pH 7.5, 150 mM KCl, 1 mM TCEP, 2 mM $MgCl_2$, 125 U DENARASE (c-LEcta), and protease inhibitor cocktail (Sigma, P8849). After 1 h incubation at 4°C, the lysate was clarified by centrifugation at 39,000 g, 30 min, 4°C. The supernatant was incubated with glutathione Sepharose 4B resin for 2 h, 4°C. After washing with lysis buffer, PHLPP2 was cleaved off the beads with TEV protease overnight. The eluate from the beads was then applied to a 1 ml HiTrap Q column (Cytiva) and PHLPP2 was eluted with a linear salt gradient. Fractions containing PHLPP2 were pooled, concentrated to 0.5 ml and applied to a Superdex 200 30/300 column equilibrated in 20 mM Tris, pH 7.5, 150 mM KCl, 1 mM TCEP. The peak fractions containing PHLPP2 were pooled, concentrated, flash frozen in liquid nitrogen, and stored at -80°C. Akt1^{3P} was prepared according to Truebestein et al, 2021 (1).

The gene sequence of *S. stenapodia* LRR-PP2C (TRINITY_DN5630_c0_g1_i3.p1) was synthesized *de novo* (Twist Bioscience) and cloned into a pFastBac Dual vector for expression in Sf9 cells. Purification followed the same procedure as human PHLPP2 with the omission of the final gel filtration step due to low protein yields. Further minor modifications include the supplementation of all buffers with 0.1 mM $MnCl_2$ and the replacement of KCl with NaCl.

Mass photometry

The mass distribution of purified PHLPP2 particles was measured on a Refeyn TwoMP mass photometer at a concentration of 25 nM. The mass of the respective peaks was estimated by fitting a Gaussian distribution to the histogram of particle masses.

RNA extraction and RNA-seq

RNA samples were extracted using the RNA isolation kit (RNAisomag) provided by the VBC Molecular Tools Shop and processed with the KingFisher Flex robot (Thermo Fisher

Scientific). For RNA sequencing, libraries were prepared using the Lexogen 3' mRNA-Seq Library Prep Kit. Sequencing was performed on a NovaSeq X 10B instrument with an XP flowcell in paired-end mode (150 bp reads) on a single lane by the NGS Facility (VBCF, Austria). FASTQ files were processed using the Nextflow 24.10.1/5930/nf-core/rnaseq v3.17.0 pipeline. Quality control was conducted with fastQC (v0.12.1). Transcript mapping to the hg38 human reference genome was performed using Salmon (v1.10.3) for pseudoalignment and STAR (v2.7.11b) as the aligner. DESeq2 (v1.42.1) was used to generate normalized count tables and perform differential expression analysis (FDR-adjusted p-value ≤ 0.05). Data visualization was performed in R using pheatmap (v1.0.12). Functional enrichment analysis was performed using enrichr (2). The RNA-seq data was uploaded to the Gene Expression Omnibus (GEO) database (accession number GSE287792).

PP2A purification

2L of yeast cells (YJV1181; gal. prom. TAP-Pph21 endogenous, BY 4741) were grown to a density of OD_{600nm}=1 in 1% raffinose and TAP-Pph21 was expressed for 4 hours by addition of 1% galactose, yielding a final cell density of OD_{600nm}=2-3. Cells were harvested by filtration and frozen in liquid nitrogen. Cells were broken up using a Freezer Mill™ cryogenic grinder (SPEX) and resuspended in 20 ml ice-cold lysis buffer (50 mM MES pH 6.5, 150 mM NaCl, 1 mM EDTA, 1% Triton X-100, Complete protease inhibitor cocktail Roche). The lysate was pre-cleared by centrifugation (15' at 16 000 g) and incubated with 300 µl IgG Sephrose™ 6 Fast Flow beads (Cytiva 17096901) for 2h at 4°C. Beads were packed into a chromatographic column and washed 1x with 20 ml lysis buffer and 3x with 20ml cleavage buffer (50 mM MES pH6.5, 150 mM NaCl, 0.1% NP40). Beads were taken up in 800 µl cleavage buffer and Pph21 was cleaved by incubating the beads for 2h at 16°C with 500 U TEV protease (NEB P8112S). Eluted Pph21 was mixed with glycerol to a final conc of 50% and stored at -20°C.

CRISPR/Cas9-mediated knockouts

HAP1 wild-type, B56β deletion (ΔB56β), and B56ε deletion (ΔB56ε) cells were obtained from Haplogene/Horizon Discovery (wild-type: #C631, ΔB56β: #HZGHC003153c009, ΔB56ε: #HZGHC003156c001). B56α was deleted in HAP1 wild-type and ΔB56ε cells

respectively by targeting exon 3 of *PPP2R5A* with CRISPR/Cas9. The guide RNA (sgRNA: 5'-AACCCACGCTTGAGGCCTCT-3') was cloned into pSpCas9(BB)-2A-Puro (PX459) V2.0 (Addgene #62988) via BbsI (B56α guide s: caccgAACCCACGCTTGAGGCCTCT, B56α guide as: aaacAGAGGCCTCAAGCGTGGGTTc) and transfected into cells using TurboFectin 8.0 (OriGene Technologies #TF81001). Co-transfection with dsRed allowed the selection of successfully transfected cells by single-cell sorting. The deletions were confirmed by western blot analysis and Sanger Sequencing. All HAP1 cell lines were cultured in GlutaMAX containing Iscove's Modified Dulbecco's Medium (IMDM) (Thermo Fisher Scientific #31980048, lot 2858891) supplemented with 10% Fetal Calf Serum (FCS) (Sigma-Aldrich #F7524, lot 0001671876) and 1% penicillin-streptomycin (Sigma-Aldrich #P4333) at 37°C and 5% CO_2.

HEK293 cell lines were cultured in DMEM high glucose (Sigma Aldrich) supplemented with 10 % FBS (Sigma Aldrich) and 1 % Penicillin/Streptomycin (Sigma Aldrich) at 37 °C and 5 % CO_2. PHLPP2 knockout HEK293 cells were generated by nucleofection of gRNA-Cas9 complexes. 150 pmol of each gRNA (AGAGAAAUAUGAAACGCAAU, AAGGACGAGAUGUAAGUCAG, Genscript) was added to 2.4 µL of RNP buffer (580 mM KCl, 40 mM Tris-HCl pH 7.5, 20 % v/v glycerol, 2 mM TCEP-HCl pH 7.5, 2 mM $MgCl_2$) and incubated for 5 min at 70 °C. 50 pmol of Cas9 (in-house purified) was added and the complex incubated for 10 min at 37°C. H_2O was added to a final volume of 10 µL. Nucleofection was performed according to the manufacturer's protocol using program CM130 (Lonza, Cell Line Nucleofector kit S). The cells were recovered for 7 days, detached and washed twice with PBS. The cells were resuspended in FACS Buffer (PBS, 10 % FBS, 1 mM EDTA) and single-cell sorted (Aurora CS) into two 96-well plates. Cells were grown to confluency and moved up to 12-well plates from which 5 x 10^5 cells were pelleted down and genomic DNA extracted using gDNA purification kit (Thermo Scientific) according to the producer´s manual. The genomic DNA samples were genotyped by PCR using the following primer combinations: AGGAGGGGATACTTGAGCAGA (upstream of KO-region) and CAGTGGTAGTGTCTGCTCCA (located inside the KO-region), or GCCCAGTGAGTCCGTAATCA (outside the cut-out region), and compared to wild-type HEK293 genomic DNA. The amplicons were gel purified in 2 % agarose gels and the deletion confirmed by Sanger sequencing.

Immunoblotting

After incubation with PHLPP2 under different conditions, the phosphorylation state of Akt1^{3P} was monitored by western blotting, using antibodies against Akt1 pT308 (Cell Signaling Technology #C31E5E) and pS473 (Cell Signaling Technology #193H12). Briefly, samples were separated by SDS-PAGE on a 12% tris-glycine gel and blotted onto nitrocellulose (0.4 μm pore size). The membrane was blocked for 1 h at room temperature with 5% BSA in 20 mM Tris, pH 7.5, 150 mM NaCl, 0.1% Tween 20. Membranes were incubated with primary antibodies at a ratio of 1:1000, overnight at 4°C, followed by washing and incubation with IRDye 800CW Goat anti-Rabbit IgG Secondary Antibody (LICOR, 926-32211) at a ratio of 1:15000, 1 h at room temperature. After washing, the membranes were imaged using a LICOR Odyssey CLx fluorescence imager.

For Akt immunoblotting in HAP1 cells, cells were grown to approx. 80% confluency, harvested and lysed in IP-Lysis buffer with protease and phosphatase inhibitors (5 mM sodium fluoride (Merck #6449), 2 mM sodium pyrophosphate (Merck #1.06591) 1 mM sodium-orthovanadate (Sigma-Aldrich #S6508), 10 mM β-glycerophosphate (Sigma-Aldrich #G6251), EDTA-free cOmplete (Roche #11836170001), Aprotinin (Sigma-Aldrich #A6279)). Lysates were cleared at 13000 g and extracted proteins were quantified with the Bio-Rad protein assay (Bio-Rad Laboratories #5000002), adjusted to equal amounts and boiled in protein sample buffer at 95°C for 5 min. Proteins were separated on 10% SDS polyacrylamide gels and transferred onto nitrocellulose membranes (GE Healthcare, 0.2μm). Membranes were stained with PonceauS and blocked using 3% skim milk (Millipore #999999-99-4, lot VM1001463145) in phosphate-buffered saline 0.05% Tween20 (PBS-T). For western blot analysis, primary phospho-specific antibodies were diluted in 5% bovine serum albumin (BSA) in Tris-buffered saline (TBS), 0.5% Tween20. All other antibodies were diluted in 0.5% skimmed milk powder in PBS-T. Antibodies [pan Akt (C67E7, Cell Signaling Technology #4691), phospho-Akt Thr308 (244F9, Cell Signaling Technology #4056), phospho-Akt Ser473 (D9E, Cell Signaling Technology #4060), B56α C-term (3A6-D3-D10-G1, Ogris Lab, directed against amino acids 463-486), B56β (6H4-3C2, Ogris Lab, directed against an N-terminal fragment encompassing amino acids 1-135), and B56ε C-term (3D1-H7-3E5-3H9, Ogris Lab, directed against amino acids 448-467)] were applied overnight at 4°C followed by incubation with horseradish peroxide-

coupled secondary antibodies directed against mouse and rabbit heavy chain IgG (Jackson Immuno Research #115-035-008, and Jackson Immuno Research #111-035-008) for 2h at room temperature. Signals were detected by incubation of the blots with Clarity Western ECL Substrate (Bio-Rad Laboratories #1705061) and subsequent imaging with the ChemiDoc Imaging System (Bio-Rad Laboratories #12003153).

Densitometric quantification was performed using the Image Lab Software. For lysate quantifications, a dilution series of the lysates was fit to a linear, exponential or logarithmic trend line by Excel. The equation of the scan with the best R^2 value was used to calculate the values of the western blot signals. Datasets were normalised to the arithmetic sum of each individual experiment (3) and means and standard deviations for each cell line were calculated. Statistical significance of immunoblotting data was assessed using one-way analysis of variance (ANOVA) and Tukey's honestly significantly different (HSD) post hoc test for multiple comparisons. In all cases, P values of <0.05 were considered to be statistically significant and are indicated with *P < 0.05, **P < 0.01, ***P < 0.005, ****P < 0.001. Statistical analysis was performed in GraphPad Prism.

Size-exclusion chromatography coupled to inductively-coupled plasma mass spectrometry

Water was purified employing an Ultra Clear basic reverse osmosis system (SGWasseraufbereitung und RegenierstationGmbH, Barsbuettel, Germany) and subsequently subboiled (MLS, Leutkirch). Formic acid Suprapure® (purity 98-100%, item no. 111670), nitric acid (purity for analysis EMSURE® Reag. Ph Eur, ISO, item no. 100456), acetic acid Suprapure® (purity 100%. Item no. 100066) and ammonia solution (purity for analysis EMSURE® Reag. Ph Eur, ISO, item no. 1005432) were purchased from Merck (KGaA, Darmstadt,Germany). Nitric acid was double subboiled and stored in perfluoralkoxy-polymer bottles. Lyophilized bovine erythrocytes Cu/Zn-superoxide dismutase (Cu/Zn-SOD, item no. S7571) was purchased from Sigma-Aldrich Chemie GmbH (Vienna, Austria).

Samples were stored at -80 °C prior to measurement. All vials and materials which came into contact with the sample, blank and standard solutions were pre-cleaned via soaking for 24 h in 10% and 24 h in 1% nitric acid followed by flushing with distilled water and drying under a class 100 laminar flow clean bench. Measurements were performed under clean room conditions (class 10000). Between the sample runs

1% formic acid solution was injected to eliminate carry over, followed by one buffer blank for assessment of possible contamination.

A NexSAR Speciation Analysis Ready HPLC system from Perkin Elmer (Massachusetts, USA) controlled by Clarity 8.8 software was used for the size exclusion separation. The mobile phase consisted of 50 mmol/L ammonium acetate at pH 7 and the flow rate was set to 0.4 mL min^{-1}. The injection volume for injection of samples, blank solutions and standards onto the ACQUITY UPLC Protein BEH 450Å SEC column (Waters™) was 15 µL. For elemental detection, the system was combined with an NexION 2000 quadrupole ICP-MS from Perkin Elmer (Massachusetts, USA). The reaction cell was filled with oxygen to induce the formation of $^{32}S^{16}O^+$ and circumvent the $^{16}O_2^+$ interference on the most abundant sulfur isotope ^{32}S. Zinc was detected as ^{66}Zn. Bovine Cu/Zn-SOD was employed to determine the intensity ratio between the ^{66}Zn and the $^{32}S^{16}O$ signals. The stoichiometric zinc ratio was calculated from the number of sulfur atoms per protein. ICP-MS parameters and data evaluation were optimized according to (4). pH measurements were conducted with an Education Line EL20 pH-meter with a micro electrode (inLab Micro, Mettler, Toledo, USA).

Phosphatase inhibitor beads (PIBs)

PIBs were prepared according to (5).

Molecular dynamics

The geometry of the predicted Zn^{2+} binding site in PHLPP2 was determined using a two-step procedure involving explicit-solvent molecular dynamics (MD) simulation followed by quantum-mechanical optimization. For MD simulations of the full-length PHLPP2, the GROMACS simulation package v. 2023 (6), the all-atom Amber99SB-ILDN force field (7) and the TIP4P-D water model (8) were used. The initial configuration of the full-length PHLPP2 protein was obtained from an AlphaFold (9) prediction for PHLPP2, including manual placement of the zinc ion in the proposed active site. To prepare the system, an energy minimization, a 10-ns constant volume equilibration (NVT) and 10-ns constant pressure equilibration (NPT) were first performed without distance restraints using the parameters given below and reaching the final all-atom RMSD with respect to the AlphaFold structure of 0.13 nm. The system was simulated in a cubic water box (8 nm x

8 nm x 8 nm) with 0.15 M NaCl to mimic experimental conditions. We also studied the influence of Zn^{2+} binding on the stability of the whole PHLPP2 structure. Due to inherent limitations, MD simulations cannot capture the quantum behavior of the metal-ion binding sites. Thus, distance restraints (k= 5000 kJ/mol/nm^2) between coordinating atoms and the zinc ion as well as between coordinating atoms themselves were included to stabilize the tetrahedral arrangement using the target distances from the X-ray structure of α-klotho (10). The restrained simulations were run for a total length of 100 ns. A leap-frog algorithm was used for integration under periodic boundary conditions. In both energy minimization and production runs, neighbor-lists were updated every 20 steps, following a Verlet-scheme based grid-search approach. The bonds involving H atoms were constrained using LINCS (11). Temperature control was achieved using a V-rescale thermostat (12), with a relaxation time of 0.1 ps, while pressure was controlled using a Parrinello-Rahman barostat (13). Compressibility for the barostat was set to 4.5 x 10^{-5} and the relaxation time was 2 ps. Coupling was done separately for water and protein in all cases. A twin-range spherical cut-off (1.0 nm/1.2 nm) was used for van der Waals interactions, while electrostatics were treated using the Particle-Mesh Ewald (PME) approach with a real space cut-off of 1.2 nm, a 0.16-nm grid and cubic interpolation. During the restrained simulation, the all-atom RMSD with respect to the to the final structure of NPT equilibration reached a stable level of 0.2-0.25 nm after 10 ns (Supplementary Figure 2F), suggesting that the placement of the zinc ion in the M2 site does not affect the structural integrity of the rest of the PHLPP2 structure. Similarly, the RMSD of coordinating atoms relative to their positions in the initial structure for the restrained simulations remains under 0.05 nm throughout the simulation (Supplementary Figure 2H). Further quantum-mechanical optimization was performed starting with a structure from the pool with the lowest 5% in binding site RMSD with respect to the equilibrated unrestrained geometry.

Quantum mechanical modeling (ONIOM)

Calculations were performed with the Gaussian 16 (Revision C.01) quantum chemistry software package (14). To reduce the computational time given the size of the system, we employed the ONIOM method (15) as implemented in the Gaussian 16 program. A two-layer QM/QM method that combines different QM methods is applied: a high-level

of accuracy for the atoms three bonds away from the zinc ion and a low level for the rest of the systems – cut from the corresponding initial structures (crystal structure of a 1:1:1 FGF23-FGFR1c-α-Klotho Ternary Complex, PDB 5W21, and MD generated model) at approximately 12 Å in radius around the metal cation where the amino acid chains logically end with methyl groups. In all calculations for the α-Klotho model system, a DFT function (M062X, B3LYP, or ωB97XD) was adopted for the inner layer, while a semi-empirical method (PM3) was used for the treatment of the real system. Different basis sets (6-31G(d,p), 6-31+G(d,p) and 6-31+G(3d,p)) in conjunction with the selected DFT functions were assessed for an accurate prediction of structural parameters (Zn-S and Zn-O bond lengths). The sulfur atom of the cysteine was modeled in its deprotonated state, in accordance with the known interaction of sulfur with zinc, which has been theoretically predicted (16) and experimentally visualized in atomic resolution crystal structures. All the methods tested predicted Zn-S bond lengths in the range 2.32-2.40 Å, and the average lengths of the three Zn-O bonds were 1.96-2.02 Å. It was concluded that both oniom(ωB97XD/6-31G(d,p):PM3) and oniom(B3LYP/6-31+G(3d,p):PM3) methods are suitable for proper description of the geometry of the α-Klotho Zn center.

Sample preparation for tandem mass spectrometry

Pellet of 1L Sf9 culture was lysed in 20 mL lysis buffer (2 mM benzamidine, 200 µL Protease inhibitor stock, 2mM MgCl2, 1 mM TCEP, 0.4 µL benzonase, 0.25% Chaps) for approximately 1 h at 4˚C in the cold room and then spun down (30 min, 18k rpm, 4˚C). The clarified supernatant was assessed for protein concentration using the Bradford assay, and two equal aliquots (1 mg/mL) were prepared. As a control, 0.5 µM okadaic acid was added to one of the samples. The two lysate samples (1 mL each) were then incubated with 20 µL of phosphatase inhibitor beads (PIBs) at 4°C for 1 hour. After incubation, the supernatant was carefully removed, and the PIBs were washed three times with 0.5 mL of Lysis buffer. Subsequently, the washed beads were incubated with 120 µL of 2% SDS for 1 hour at 65˚C and the eluates collected using Pierce Micro-Spin columns.

The samples were mixed with 5µL 100mM Tris HCl pH 8.5 and heated at 95°C for 5min. Then disulfide bonds were reduced with 4 µL of 250 mM dithiothreitol (DTT) for 30 min at room temperature before adding 4 µL of 500 mM iodoacetamide and incubating for 30 min at room temperature in the dark. The remaining iodoacetamide was quenched

with 2 µL of 250 mM DTT for 10 min. Sera-MagSpeed Beads mix (GE Life Sciences, cat. nos. 45152105050350 and 65152105050350) was added to the sample at beads:protein ratio of 10:1 and mixed with ethanol to reach 50% final ethanol concentration. The tubes were kept in a thermomixer for 10min at 1000rpm for 24min to let protein bind to the beads. Then the supernatant was removed using a magnetic rack and the beads were washed with 180 µL 80% ethanol in water off the rack. And the wash procedure was repeated 3 more times. Afterwards, 30µL 50mM ammonium bicarbonate with 120 ng trypsin (Trypsin Gold, Promega) was added to the beads and incubated at 37°C 1000rpm overnight. The beads were settled on a magnetic rack and the supernatant was transferred to a new tube. The beads were rinsed with 30µL ammonium bicarbonate and sonicated for 30s and the supernatant was pooled to the supernatant from the previous step. Then the samples were centrifuged at 20000g for 1min and the supernatant was transferred to a new tube to remove any remaining beads. Trifluoroacetic acid was added to reach a final concentration of 0.5%.

For the analysis of recombinant PHLPP2, ~5 µg protein (5 µL) was mixed with 15 µL 8M urea 50mM ammonium bicarbonate. Disulfide bonds were reduced with 0.8 µL of 250 mM dithiothreitol (DTT) for 30 min at room temperature before adding 0.8 µL of 500 mM iodoacetamide and incubating for 30 min at room temperature in the dark. The remaining iodoacetamide was quenched with 0.4 µL of 250 mM DTT for 10 min. 50mM ammonium bicarbonate was added to dilute the urea to 1M. The protein was digested with 160 ng trypsin (Trypsin Gold, Promega) at 37°C overnight. The digest was stopped by adding trifluoroacetic acid to reach a final concentration of 0.5% and the peptides were desalted using C18 Stagetips (17).

For the differential alkylation experiment purified PHLPP2 was incubated in 5 mM iodoacetamide for 30 minutes in the dark alkylating accessible thiol groups. Unreacted iodoacetamide was removed by buffer exchange to 50 mM ammonium bicarbonate using Bio-Rad Micro Bio-Spin™ columns. Solid urea was added to the eluate to reach a concentration > 6 M urea. Subsequently disulfide bridges were reduced adding tris (2-carboxyethyl) phosphine (TCEP) to a final concentration of 1 mM and incubated for 30 minutes. Free thiols were then modified with methyl methanethiosulfonate (MMTS) at a concentration of 5 mM. After diluting to 1 M urea with 50 mM ammonium bicarbonate the sample was digested with trypsin at a protease:protein ratio of 1:30 at 37°C

overnight. The digest was stopped by adding trifluoroacetic acid to reach a final concentration of 0.5 % and the peptides were desalted using C18 Stagetips.
As reference samples, the protein was denatured in 6.4M urea, reduced in 1mM TCEP and free thiols alkylated either with 5 mM iodoacetamide or 5 mM MMTS for 30 minutes in the dark prior digest and clean-up as described above.

Liquid chromatography- tandem mass spectrometry

Peptides were separated on an Ultimate 3000 RSLC nano-flow chromatography system (Thermo-Fisher), using a pre-column for sample loading (Acclaim PepMap C18, 2 cm × 0.1 mm, 5 µm, Thermo-Fisher), and a C18 analytical column (Acclaim PepMap C18, 50 cm × 0.075 mm, 2 µm, Thermo-Fisher), applying a segmented linear gradient from 2% to 35% and finally 80% solvent B (80% acetonitrile, 0.1% formic acid; solvent A 0.1% formic acid) at a flow rate of 230 nL/min over 60min . Eluting peptides were analyzed on an Exploris 480 Orbitrap mass spectrometer (Thermo Fisher), which was coupled to the column with a FAIMS pro ion-source (Thermo Fisher) using coated emitter tips (PepSep, MSWil).

For analysis of peptides obtained from PIB eluates, the mass spectrometer was operated in DIA mode with the FAIMS CV set to -45, the survey scans were obtained in a mass range of 350-1200 m/z, at a resolution of 60k at 200 m/z and a normalized AGC target at 300%. 31 MSMS spectra with variable isolation width between 13 and 24 m/z covering 349.5-1200.5 m/z range including 1 m/z windows overlap, were acquired in the HCD cell at 30% collision energy at a normalized AGC target of 1000% and a resolution of 30k. The max. injection time was set to auto.

For analysis of peptides obtained from purified, recombinant PHLPP2, the mass spectrometer was operated in DDA mode with two FAIMS compensation voltages (CV) set to -45 or -60 and 1 s cycle time per CV. The survey scans were obtained in a mass range of 350-1500 m/z, at a resolution of 60k at 200 m/z, and a normalized AGC target at 100%. The most intense ions were selected with an isolation width of 1.2 m/z, fragmented in the HCD cell at 28% collision energy, and the spectra recorded for max. 100 ms at a normalized AGC target of 100% and a resolution of 15k. Peptides with a charge of +2 to +6 were included for fragmentation, the peptide match feature was set to

preferred, the exclude isotope feature was enabled, and selected precursors were dynamically excluded from repeated sampling for 20 seconds.

For the differential alkylation experiments, the Exploris 480 Orbitrap mass spectrometer was run without FAIMS. The survey scans were obtained in a mass range of 350-1500 m/z, at a resolution of 60k at 200 m/z, and a normalized AGC target at 300%. The 10 most intense ions were selected with an isolation width of 1.2 m/z, fragmented in the HCD cell at 30% collision energy, and the spectra recorded for max. 100 ms at a normalized AGC target of 200% and a resolution of 15k.

MS data analysis

Raw data were processed using Spectronaut software (version 18.3, https://biognosys.com/software/spectronaut/) with the DirectDIA+ workflow or FragPipe (version 19.1) (18). The Uniprot Spodoptera_frugiperda proteome (version 2023.03), PHLP2_HUMAN protein sequence, as well as a database of most common contaminants were used. The searches were performed with full trypsin specificity and a maximum of 2 missed cleavages at a protein and peptide spectrum match false discovery rate of 1%. Carbamidomethylation of cysteine residues were set as fixed, oxidation of methionine and N-terminal acetylation as variable modifications. The cross-run normalization was turned off and all other settings were left as default. For the differential alkylation analysis, modification of cysteines with iodoacetamide or MMTS were defined as variable modifications.

Computational analysis was performed using Python and the in-house developed Python library MsReport (version 0.0.19) (19). Protein intensity less than 1000 was set to missing to remove the low-quality quantification values. LFQ protein intensities reported by SpectroNaut were log2-transformed and normalized across samples using the ModeNormalizer from MsReport. The missing normalized LFQ intensity values were imputed by drawing random values from a normal distribution after filtering out contaminants, proteins with less than 2 peptides and less than 1 quantified values in at least one group. Using MsReport, iBAQ intensities were calculated from the raw intensities reported by FragPipe.

The proteomics data have been deposited to the ProteomeXchange Consortium via the PRIDE partner repository (20) with the dataset identifiers PXD045891, PXD045978 and PXD052551.

Immunoprecipitation of PHLPP2

HEK293 cells ($5x10^6$) were transiently transfected with a plasmid encoding N-terminally V5-tagged PHLPP2 using Lipofectamine 3000 according to the manufacturer's protocol. Cells were harvested after two days, lysed with 3 repeated freeze/thaw cycles in liquid nitrogen, and then resuspended in 500 µL lysis buffer: 50 mM Tris pH 7.5, 150 mM KCl, 1 mM TCEP, 0.25% CHAPS, 2 mM $MgCl_2$, 2 mM benzamidine, supplemented with 0.01 µL DENARASE® and 5 µL protease inhibitor cocktail, (Sigma P8849).

The lysate was combined with 25 µL of V5-trap magnetic beads (Chomotek v5tma) pre-equilibrated in wash buffer (50 mM Tris pH 7.5, 150 mM KCl, 1 mM TCEP) and incubated for 3 h at 4°C on a rotary plate. A magnetic rack was used to pull down the beads and the supernatant (lysate) was removed. The beads were resuspended in wash buffer (beads). The wash step was repeated 5 times. Samples of the lysate, beads, and supernatant were taken after each wash step. All samples were subjected to a malachite green assay (Sigma-Aldrich MAK307) as described in the Methods section of the manuscript.

For western blotting, the samples were resolved by SDS-PAGE on a 12% Tris-Glycine gel. Transfer onto a 0.2 µm nitrocellulose membrane was performed at 120 V and 4°C for 2.5 h. The membrane was incubated with 5 % BSA in TBST for 1 h while gently shaking. V5-Tag rabbit monoclonal antibody (Cell Signaling, #13202) and β-actin mouse monoclonal antibody (Cell Signaling, #3700) were diluted 1:1000 in 3% BSA in TBST to a final volume of 5 mL and incubated with the membrane overnight at 4°C. The membrane was washed 3 times with TBST for 5 min each. Goat anti-rabbit secondary antibody (LI-COR, 926-32211) and goat anti-mouse secondary antibody (LI-COR, 926-68070) were diluted 1:15000 in 3% BSA in TBST to a final volume of 5 mL and incubated with the membrane in the dark for 1 h at room temperature while gently shaking. The membrane was washed 3 times with TBST for 5 min each and imaged using a LI-COR Odyssey CLx fluorescence imager.

AlphaFold2 structure prediction

The structures of PHLPP2 and Cyr1 homologs, as well as the Cyr1 dimer and complexes of Cyr1 with RAS1/RAS2 and CAP were predicted with AlphaFold2 multimer_v3 (9) installed on a local cluster. The top five models output in each case were compared to each other for convergence by calculating the r.m.s.d. over equivalent Ca atoms and evaluated according to their respective pair alignment error plots (PAE).

Cryo-electron microscopy

Grid preparation. Open-hole cupper 200 mesh grids overlayed with R 2/2 carbon film (Quantifoil) were used for plunge freezing. Grids were glow-discharged with a BalTec SCD 005 sputter coater for 60 s at 25 mA using residual air. 4 µl of the sample at concentration of 0.1 mg/ml was applied onto the carbon side and front-side blotted using the Leica GP2 plunge freezer. The sample was blotted for 2 seconds including the integrated blot sensor.

Data collection. Initial grid screening was performed on a 200kV Glacios microscope (ThermoFisher) using a Falcon III detector (ThermoFisher). For high-resolution structure determination, data collection was performed at Diamond Light Source Krios I using a K3 detector (Gatan). Images were collected at super-resolution pixel size of 0.53 Å/pix with a cumulative fluence of 35 $e^{-}/Å^{2}$ in a defocus range from 1.7 to 2.5 µm in 0.2µm increments using EPU (ThermoFisher).

Image processing. Images were preprocessed using the cryosparc (21) live pipeline. In short, micrograph movies were aligned, fourier binned by a factor of two and the CTF parameters were determined. Particles were picked initially via blob picking. Clustering these results in 2D led to new templates that have been subsequently been used for particle selection. Particles underwent 2 rounds of 2D clustering and an ab-initio model was generated and refined. These preprocessed particles were transferred to relion 5 (22). 3D Clustering generated two classes with distinct features but only one was refining to subnanometer resolution. Refinement was performed using Blush regularisation (23).

Modelling. Into the map an alphafold (9) model was placed using UCSF ChimeraX (24). Models were adjusted and refined using MDFF in Namdinator (25).

Phylogenetic and sequence analysis

To examine the evolutionary history of PHLPP, we generated a database of genome-predicted eukaryotic reference proteomes from UniProt (*n* = 181, downloaded 25 January 2024) (26). This database was taxonomically balanced by selecting the best two proteomes per genus based on BUSCO (Benchmarking Universal Single Copy Orthologues) completeness (27). For metazoans, fungi, and embryophytes, more strict taxonomic criteria were set by selecting the best proteome per phyla for metazoans (*n* = 17), the best proteome per class for fungi (max two per phylum, *n* = 12), and the best proteome per order for embryophytes (max three per class, *n* = 11). To identify PHLPP homologs, the database was searched using Diamond BLASTp v2.0.9 with human PHLPP1 (UniProt accession: O60346) as a query (query coverage ≥ 25%, $E < 10^{-5}$, sensitive mode) (28). The resulting hits were aligned using MAFFT v7.520 trimmed using trimAl v1.4.rev15 with a gap threshold of 50%, and a maximum-likelihood phylogeny was inferred with IQ-Tree v2.2.6 using the LG4M substitution model with topology support assessed using Shimodaira-Hasegawa approximate likelihood ratio tests (SH-aLRT, *n* = 1,000) and ultra-fast bootstraps (*n* = 1,000) (29–33). The phylogenies were inspected manually in FigTree v1.4 (http://tree.bio.ed.ac.uk/software/figtree/) and paralogs were excluded. The resulting homologs were then used for two additional iterative BLAST searches with stricter E-values of 10^{-25}, and the hits were analyzed phylogenetically and curated as described. To improve taxonomic sampling, we conducted an additional search using the EukProt V3 database and incorporated the resulting hits to produce a complete set of LRR-PP2C homologues from diverse eukaryotes (34).

Phylogenetic analyses were used to reconstruct the evolutionary history of PHLPP. To this end, the identified PHLPP homologues were aligned with MAFFT using the high accuracy L-Ins-i algorithm and the alignment was trimmed as before. A maximum likelihood phylogeny was inferred using the LG4M+F+R7 substitution model which was selected based on Bayesian information criteria using ModelFinder (35). Statistical support was inferred as before using SH-aLRT and the phylogeny was rooted

based on recent eukaryotic species phylogenies (36). The monophyly of the unicellular holozoans was assessed using an approximately unbiased (AU) test implemented in IQ-Tree using a constrained phylogeny inferred under the same model as the unconstrained tree (37). The phylogenies were visualized and annotated using iTOL v6 (38).

Protein domain annotations were accomplished by mapping profile hidden Markov models (HMM) obtained from the Pfam-A database to protein sequences using HMMER v3.4 ($E < 10^{-3}$) (39, 40). However, to improve HMM sensitivity, the mapped domains were extracted, aligned using MAFFT, and used to produce new HMMs. This process was repeated iteratively three times until consistent domain annotations were obtained. Active site residue conservation was investigated using sequence logos generated with Skylign using weighted counts (41).

Lastly, to identify the origin of the unicellular holozoan kinase domains, the reference database was searched using Diamond BLASTp ($E < 10^{-5}$, query coverage ≥ 50%, sensitive mode, max 250 targets per query sequence) and the holozoan kinases as a query. The kinase domains of the resulting proteins were excised and subsequently aligned with the PHLPP kinases using MAFFT L-Ins-i. The alignment was then trimmed using trimAl with a gap threshold of 50% and a phylogeny was generated using IQ-Tree as described above, using the LG4M+I+R10 substitution model selected with ModelFinder. Proteins were annotated based on BLAST searches against SWISS-PROT (42).

Phylogenomic profiling

To examine the distribution of PHLPP paralogs across the gnathostome phylogeny an additional database of representative craniate species was assembled using predicted reference proteomes from UniProt (downloaded 12 November 2024). PHLPP1 and PHLPP2 along with AKT and PKC orthologues were identified across craniate species as described above. When orthologues were missing, previously identified orthologues were used as queries for tBLASTn ($E < 10^{-5}$) searches against animal genomes and protein predictions were inferred using Exonerate v2.2(see https://github.com/nickatirwin/Phylogenomic-analysis; (43) and phylogenetically curated. To identify proteins with correlated distributions, craniate

proteomes were clustered in hierarchical orthogroups (N0) using OrthoFinder v2.5.5 (using ultra-sensitive Diamond and FastTree for tree inference). Co-evolutionary patterns were detected based on previously established methods (44). In brief, for each gene, orthologue presence was recoded in binary and, to account for taxonomic sampling, median values were inferred for each clade containing or lacking PHLPP2. Orthologoues found in less than 30 species were excluded and the associations between PHLPP2 and each orthologue were inferred using point biserial correlations, given the binary nature of PHLPP2 presence. Statistical significance was inferred using permutation tests by recreating orthogroups by randomly sampling sequences across all proteomes without replacement and re-calculating correlations (n = 10,000, $p < 0.05$). Positively correlated clusters were further refined through additional homology searches and manual phylogenetic curation.

Lipid-protein overlay assay

PIP strips (P-6001) were purchased from Echelon Biosciences and a lipid-protein overlay assay was performed according to the manufacturer's protocol in duplicates. In brief, the membrane was blocked with 3% lipid-free BSA in PBS-T. GST-tagged recombinant PHLPP2 was added to a final concentration of 1.8 μg/mL in PBS-T and incubated for 1 h at 25 °C. The membrane was washed three times with PBS-T and subsequently incubated with primary mouse anti-GST antibody (Invitrogen, MA4-004) at 25 °C for 1h. The membrane was washed three times with PBS-T followed by an incubation with secondary goat anti-mouse antibody (Licor, IRDye 680RD Goat anti-Mouse IgG Secondary Antibody). The membrane was washed three times with PBS-T and imaged using a LI-COR Odyssey CLx fluorescence imager.

Supplementary Figure Legends

Supplementary Figure 1. Biochemical and biophysical analysis of purified PHLPP2.

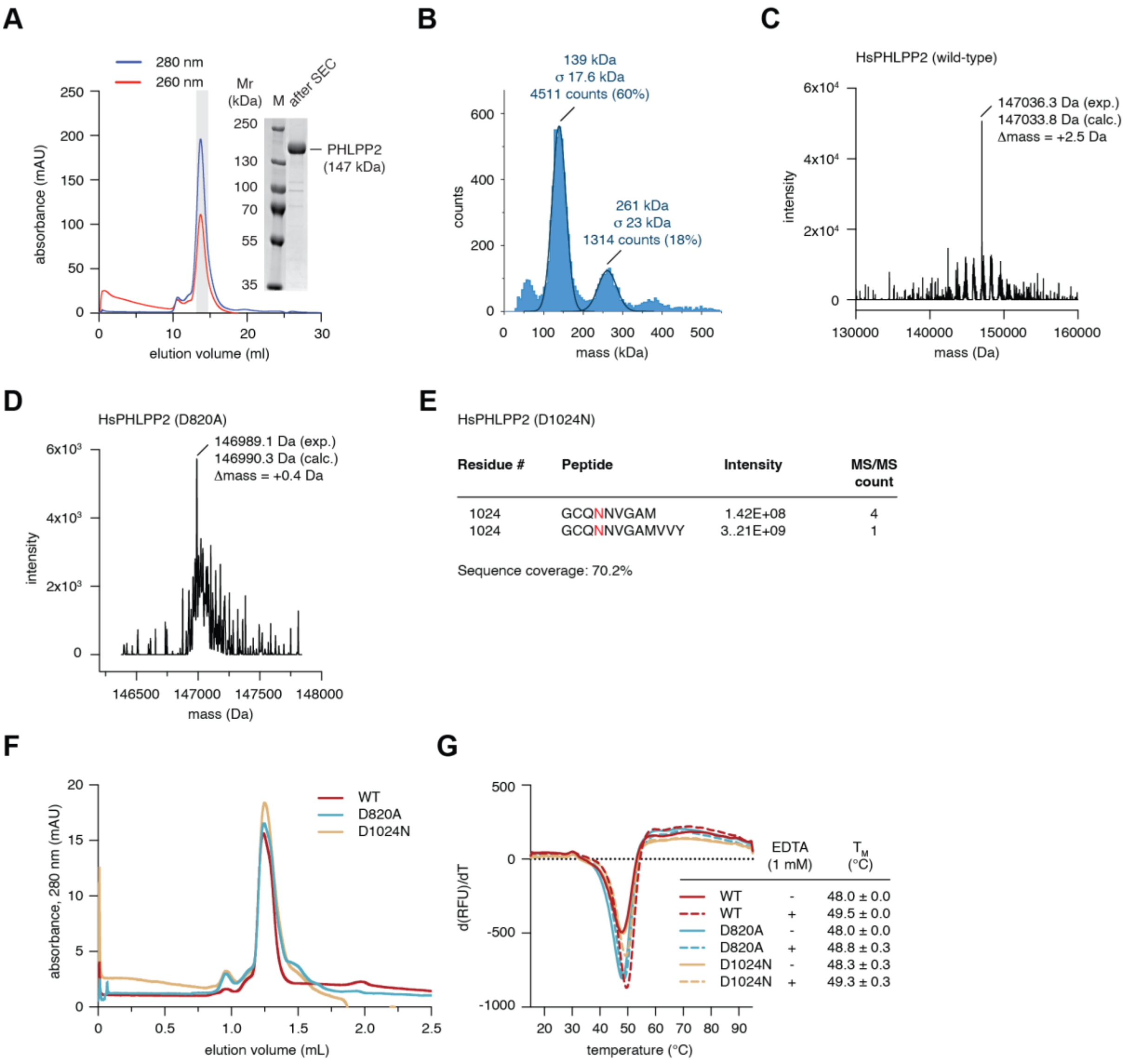

A. Size-exclusion chromatography of purified, recombinant human PHLPP2.

B. Mass photometry of recombinant PHLPP2.

C. Mass spectrum of recombinant human PHLPP2^{WT}.

D. Mass spectrum of recombinant human PHLPP2^{D820A}.

E. Peptide fingerprinting of recombinant human PHLPP2^{D1024N}. The -1 Da mass difference of the mutant was detected by tandem mass spectrometry analysis of chymotryptic peptides.

F. Analytical size exclusion chromatography of wild-type PHLPP2, PHLPP2^{D820A} and PHLPP2^{D1024N}.

G. Thermal stability measurements of wild-type PHLPP2, PHLPP2^{D820A} and PHLPP2^{D1024N} in the presence and absence of 1 mM EDTA.

Supplementary Figure 2. PHLPP2 exhibits no detectable activity against Akt.

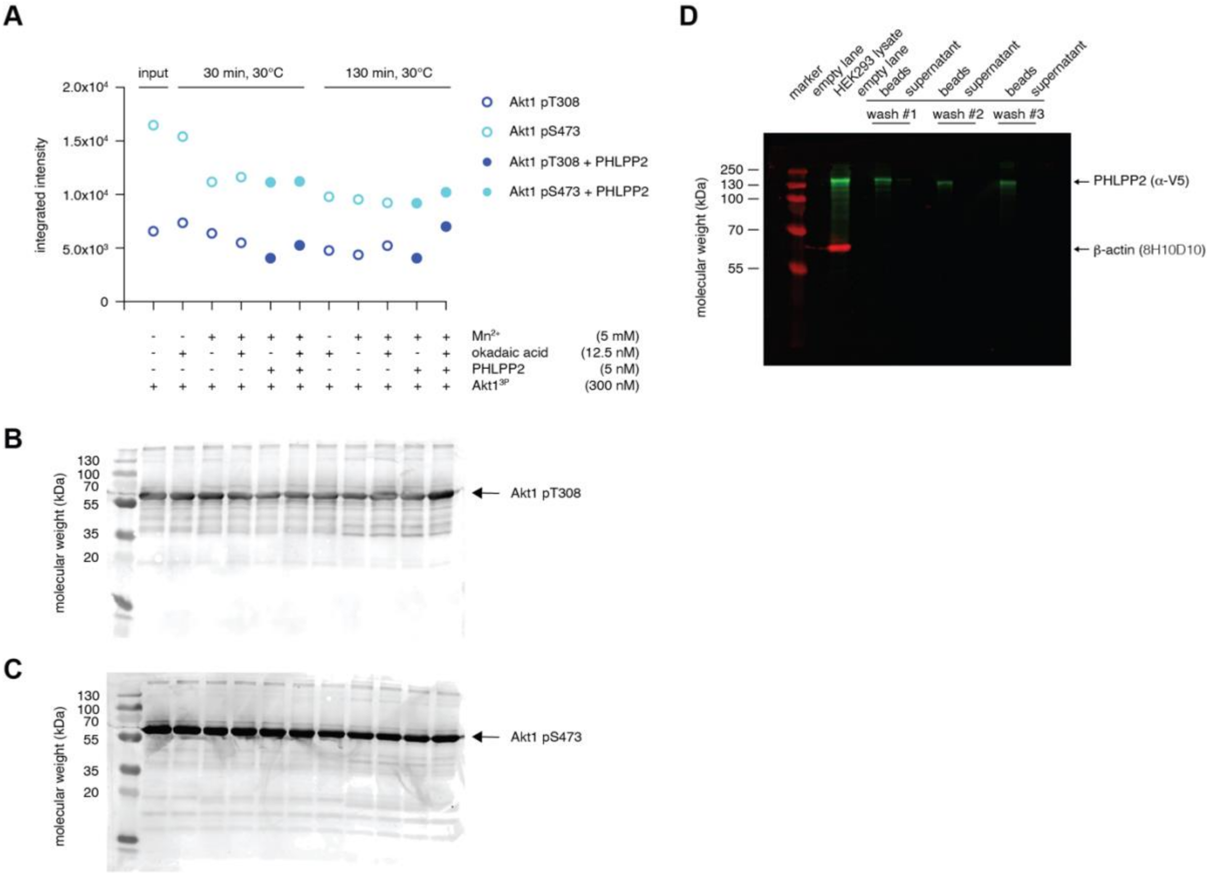

A. Akt1^{3P} dephosphorylation by purified PHLPP2, according to the assay conditions of Gao et al (45). Quantification of western blots shown in panels B and C.

B. Western blot of Akt1 pT308.

C. Western blot of Akt1 pS473.

D. Western blot of samples analyzed for phosphatase activity in Figure 4H. Immunoblotting was carried out with mouse anti-β-actin antibody and a rabbit anti-V5 antibody followed by detection with AlexaFluor-647 anti-mouse IgG and AlexaFluor-488 conjugated anti-rabbit IgG secondary antibodies respectively.

Supplementary Figure 3. Akt-FOXO1 signaling is unaffected by PHLPP2 knockout.

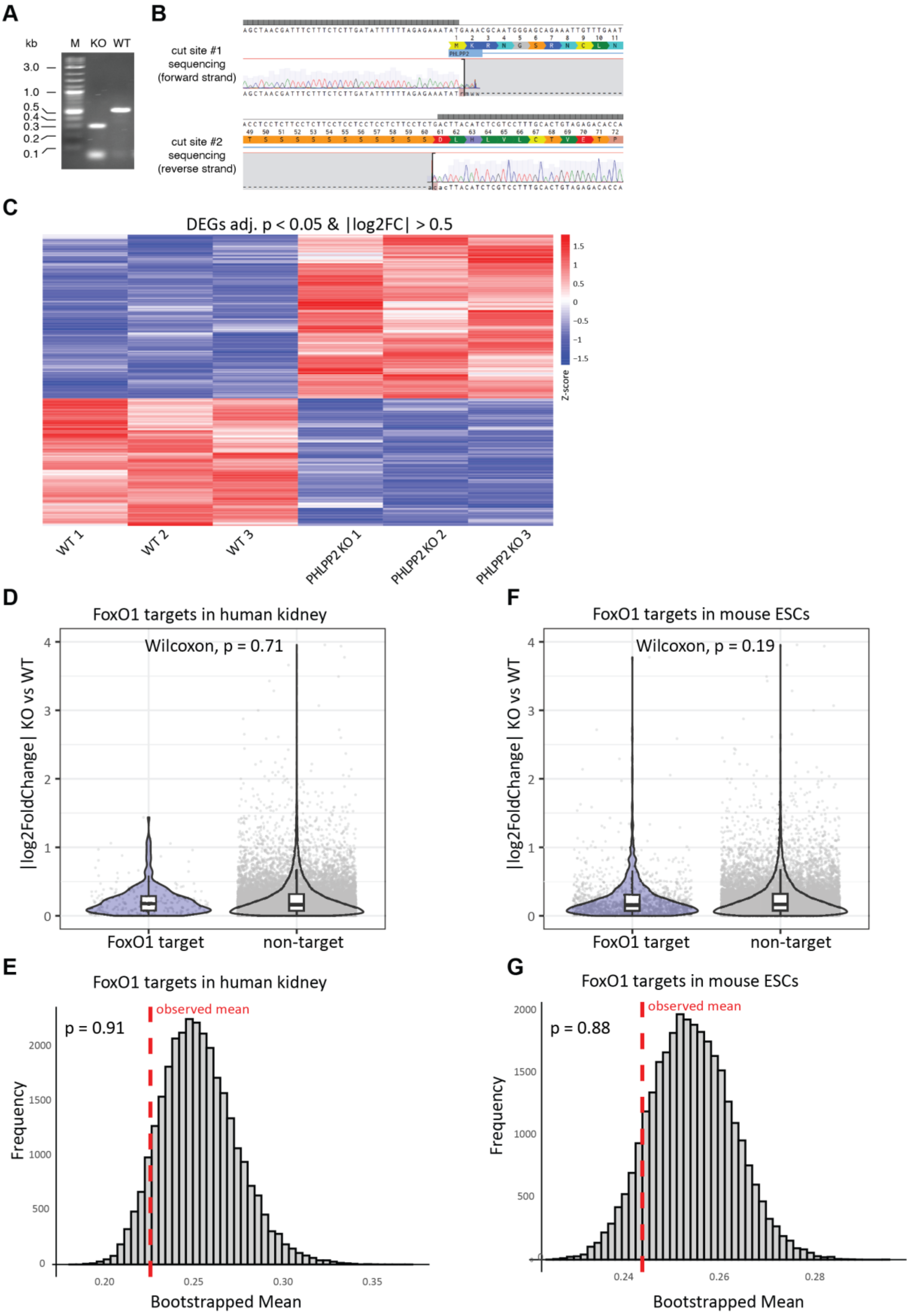

A. PCR genotyping of PHLPP2 KO compared to WT HEK293 cells.

B. Sanger sequencing of deletion in PHLPP2 KO cells.

C. Heatmap showing Z-transformed normalized counts of genes identified as significantly differentially expressed between PHLPP2 KO and WT in all replicates.

D. Plot showing the absolute log2FCs (PHLPP2 KO vs WT) of FOXO1 targets in kidney compared to non-targets in the PHLPP2 KO RNA-seq dataset. The *p*-value indicates significance.

E. Bootstrapping analysis. 50,000 random draws of 367 genes (=number of FOXO1 targets in kidney) from the PHLPP2 KO RNA-seq dataset. The distribution of average log2FC (PHLPP2 KO vs WT) is plotted, with *p*-value indicating the probability of the predicted exceeding the measured effect-size.

F. Similar to D with plot showing the absolute log2FCs (PHLPP2 KO vs WT) of FOXO1 targets in mouse embryonic stem cells and in early differentiation compared to non-targets in the PHLPP2 KO RNA-seq dataset. p-values indicate significance.

G. Similar to C with draws of 2513 gens (=number of FOXO1 targets in mouse embryonic stem cells and in early differentiation).

Supplementary Figure 4. Phosphatase inhibitor beads selectively enrich PP2A, PP4 and PP6.

A

Leading protein	Gene name	Protein name	Organism	Molecular weight (kDa)	Total # unique peptides	Sequence coverage	Imputed LFQ intensity [log2] -OA	Imputed LFQ intensity [log2] +OA (ctrl)	Enrichment ratio [log2]
A0A2H1WUX1	SFRICE_008486	60S ribosomal protein L24	*Spodoptera frugiperda*	17.39	2	13.50	25.37	15.00	10.37
J9Z496		PP2A C-subunit		35.44	23	68.30	25.69	15.46	10.23
A0A2H1W5F9	SFRICE_005114	Abl tyrosine kinase		63.75	2	4.80	20.68	10.74	9.94
A0A2H1X028	SFRICE_014915	PP2A A-subunit		65.27	39	68.00	27.03	17.94	9.08
A0A2H1WQP4	SFRICE_007634	PP2A 56 kDa γ-like subunit*		9.37	2	31.80	17.65	9.20	8.45
A0A2H1VFZ6	SFRICE_004402	Protein Phosphatase 4 C-subunit		35.53	10	27.70	23.37	15.53	7.84
A0A2H1WYQ1	SFRICE_025240	PP2A 56 kDa ε regulatory subunit		41.67	15	37.50	20.99	13.68	7.31
A0A2H1W1C4	SFRICE_000120	uncharacterized protein		20.10	3	17.70	16.82	9.94	6.88
A0A2H1V7H1	SFRICE_001858	Striatin-interacting protein (STRIPAK)		141.61	22	19.10	20.80	14.26	6.53
A0A2H1VCR1	SFRICE_002609	Striatin-3 (PP2A B-subunit)		75.12	30	54.30	22.48	16.20	6.28
A0A2H1V3Y5	SFRICE_015530	PP2A 55 kDa regulatory subunit B		56.78	24	45.10	23.73	17.53	6.20
A0A2H1WL05	SFRICE_022984	MOB kinase activator-like 4		25.51	9	36.70	22.69	16.74	5.95
A0A2H1VWP9	SFRICE_003851	Protein phosphatase 6 regulatory subunit 3-A		91.52	17	20.10	23.07	17.66	5.42
A0A2H1WE70	SFRICE_008170	Protein phosphatase 4 regulatory subunit 3		90.35	23	31.30	18.20	13.49	4.71
A0A2H1V4G9	SFRICE_023010	Myelin P2 protein		21.00	2	10.60	18.11	13.77	4.34
A0A2H1X0P5	SFRICE_029750	Protein phosphatase 6 C-subunit		34.86	13	37.60	22.00	17.74	4.26
A0A2H1W001	SFRICE_003894	Protein phosphatase 4 regulatory subunit 2		54.77	20	46.30	19.80	15.59	4.21
A0A2H1X3N4	SFRICE_031227	Microtubule-associated protein RP/EB 1		41.94	2	4.80	15.15	10.99	4.16
Q6ZVD8	Q6ZVD8	PHLPP2 (heterologous over-expression)	*Homo sapiens*	146.66	19	17.10	19.16	19.68	-0.53

A. Tandem mass spectrometry analysis of proteins bound to microcystin-LR-conjugated beads after incubation with cell lysates of Sf9 cells heterologously over-expressing PHLPP2. Red: protein phosphatases and protein-phosphatase-associated regulatory proteins.

Supplementary Figure 5. PP2A B56β dephosphorylates Akt in HAP1 cells.

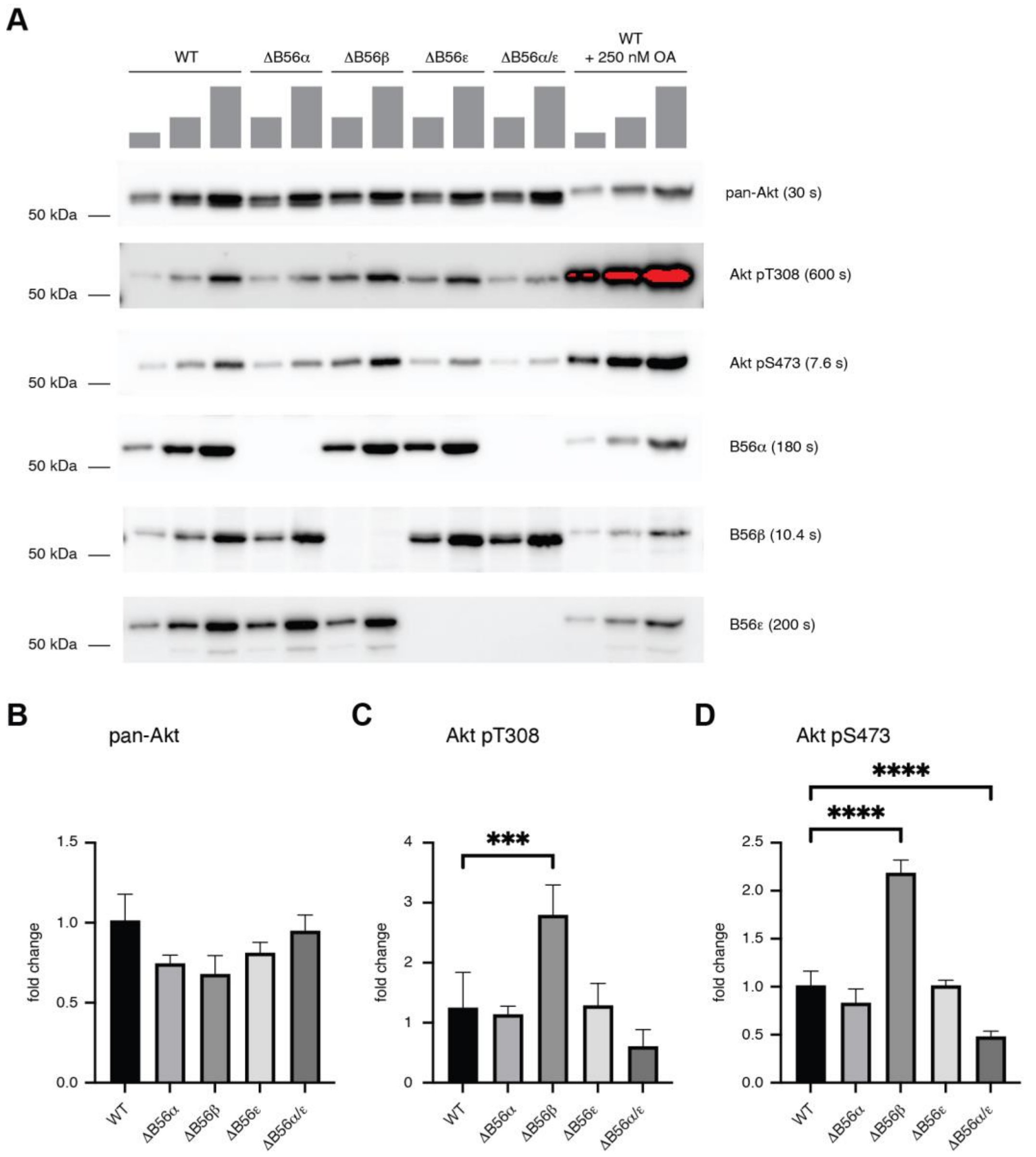

A. Immunoblotting of HAP1 wild-type, ΔB56α, ΔB56β, ΔB56ε, ΔB56α/ε, and okadaic acid treated (250nM for 3h) wild-type cell lysates using indicated antibodies. The blots are representative of n=4 experiments. Signals obtained from pan-Akt, Akt pT308, Akt pS473 were quantified, and Akt pT308 and Akt pS473 were normalised to pan-Akt. The data is shown as mean values ± s.d. relative to the wild-type, set to 1. Statistical significance was assessed by performing an ordinary one-way ANOVA. *$P < 0.05$ ***$P < 0.005$, ****$P < 0.001$.

B. Quantification of pan-Akt signal (n=4).

C. Quantification of Akt pT308 signal (n=4).

D. Quantification of Akt pS473 signal (n=4).

Supplementary Figure 6. PHLPP2 is a zinc-binding protein.

A

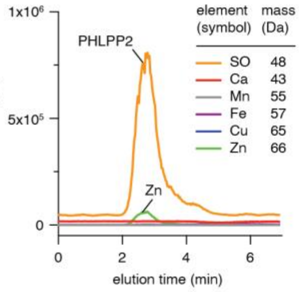

B

Sample	^{32}S peak area (counts)	^{65}Cu peak area (counts)	^{66}Zn peak area (counts)	S/Cu ratio	S/Zn ratio	stoichiometry (S:Cu:Zn)
**SOD**[1] (calibrant)	1324513	2253245	1854913	0.5878	0.7141	4:1:1
	1321345	2249440	1826849	0.5874	0.7233	
	1359483	2313953	1880422	0.5875	0.7230	
Average				0.5876	0.7201	
RSD (n=3)				<1%	<1%	
**PHLPP2**[2] (replicate #1)	50032296	448795	3674225	111.4815	13.6171	67 : 0.09 : 0.88
	48727540	415543	4204563	117.2623	11.5920	67 : 0.08 : 1.04
	18592408	111698	1685312	166.4532	11.0320	67 : 0.06 : 1.09
Average						67 : 0.08 : 1.01
RSD (n=3)	Total combined uncertainty of the measured stoichiometry: 11%					
**PHLPP2**[2] (replicate #2)	38036679	-	2752163	-	13.8207	67 : - : 1.09
	23377197	-	1600115	-	14.6097	67 : - : 1.03
	55531332	-	4939412	-	11.2425	67 : - : 1.35
Average						67 : - : 1.16
RSD (n=3)	Total combined uncertainty of the measured stoichiometry: 14%					
**PHLPP2**[2] (replicate #3)	350283355	-	35552871	-	9.8525	67 : - : 1.25
	403417464	-	42803314	-	9.4249	67 : - : 1.31
Average						67 : - : 1.28
RSD (n=2)	Total combined uncertainty of the measured stoichiometry: 10%					

[1]Bovine SOD1; 3 Cys + 1 Met = total 4S.
[2]Human PHLPP2; 40 Cys + 27 Met = total 67S.

C

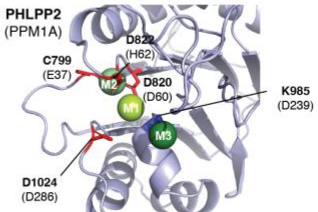

D

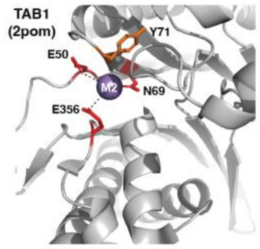

F

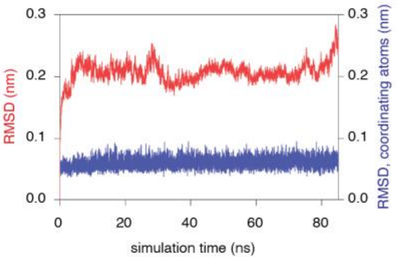

G

ONIOM validation (α-klotho)	Bond: S - Zn^{2+} (Å)	Bond: O - Zn^{2+} (Å)
experimental structure (5W21)	2.30	1.99
B3LYP/6-31G(d,p):PM3	2.34	1.97
B3LYP/6-31+G(d,p):PM3	2.36	2.02
B3LYP/6-31+G(3d,p):PM3	2.34	2.01
MO62X/6-31G(d,p):PM3	2.39	1.99
MO62X/6-31+G(d,p):PM3	2.39	2.00
MO62X/6-31+G(3d,p):PM3	2.40	2.01
ωB97XD/6-31G(d,p):PM3	2.33	2.96
ωB97XD/6-31+G(d,p):PM3	2.32	2.01

E

Residue # (CYS)	Structure prediction	coverage (Y/N)	MMTS/IAA ratio	solvent accessible / inaccessible?
133	solvent accessible (RA)	Y	0.23	accessible
143	solvent accessible loop (RA)	Y	0.01 - 0.02	accessible
177	PH-LRR interface	Y	n.d.	n.d.
180	PH-LRR interface	Y	n.d.	n.d.
189	solvent accessible (PH)	Y	0	accessible
258	solvent accessible (RA-LRR)	Y	0.27	accessible
402	solvent accessible (LRR)	Y	0.06	accessible
460	solvent accessible (LRR)	Y	n.d.	n.d.
467	core (LRR)	Y	25.46 - 41.28	inaccessible
516	solvent accessible (LRR)	Y	0	accessible
522	core (LRR)	Y	3.67	partially accessible
690	core (LRR)	Y	17.94 - 34.16	inaccessible
799	solvent accessible (PP2C)	Y	10.62	inaccessible
833	solvent accessible (PP2C)	Y	0.05 - 0.07	accessible
878	core (PP2C)	Y	n.d.	n.d.
901	core (PP2C)	Y	n.d.	n.d.
906	core (PP2C)	Y	n.d.	n.d.
948	solvent accessible (PP2C)	Y	6.5	
949	solvent accessible (PP2C)	Y	9.21	
955	solvent accessible (PP2C)	Y	0.47 - 3.38	partially accessible
1118	solvent accessible (C-ter.)	Y	2.83 - 2.87	partially accessible
1173	solvent accessible (C-ter.)	Y	0.13 - 0.22	accessible
1174	solvent accessible (C-ter.)	Y	0.13 - 0.22	accessible
1193	solvent accessible (C-ter.)	Y	0.14 - 1.45	accessible
1202	solvent accessible (C-ter.)	Y	0.04 - 0.10	accessible
1234	solvent accessible (C-ter.)	Y	0.00 - 0.01	accessible
9	solvent accessible (N-ter.)	N	-	-
32	core (RA)	N	-	-
67	core (RA)	N	-	-
77	core (RA)	N	-	-
319	core (LRR)	N	-	-
331	solvent accessible (LRR)	N	-	-
351	solvent accessible (LRR)	N	-	-
613	solvent accessible (LRR)	N	-	-
636	core (LRR)	N	-	-
722	LRR-PP2C interface	N	-	-
1014	core (PP2C)	N	-	-
1022	core (PP2C)	N	-	-
1040	solvent accessible (C-ter.)	N	-	-
1042	solvent accessible (C-ter.)	N	-	-

n.d. = not determined

H

Residue	Bond	Molecular Dynamics (Å)	Quantum Mechanics: B3LYP/ 6-31+G(3,d,p):PM3 (Å)	Quantum Mechanics: ωB97XD/ 6-31G(d,p):PM3 (Å)
C799	S - Zn^{2+}	3.0	2.34	2.33
D820	O - Zn^{2+}	1.8	2.04	1.96
D822	O - Zn^{2+}	1.9	2.03	1.97
D1024	O - Zn^{2+}	1.8	1.99	1.94

I

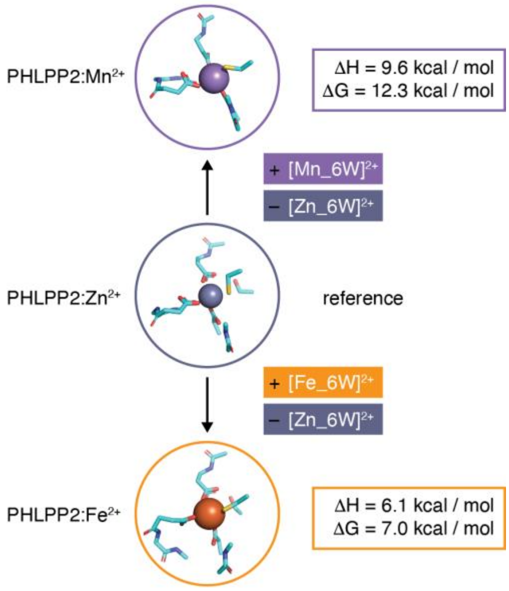

A. ICP-MS chromatograms for recombinant human PHLPP2, including the sulfur signal from the protein.

B. Calibration of PHLPP2:Zn^{2+} stoichiometry for three biological replicates, each with duplicate or triplicate technical replicates.

C. Metal ion positions of PPM1A (PDB ID: 6b67) superimposed on AF2 model of PHLPP2 PP2C domain.

D. Experimentally determined structure of TAB1 pseudophosphatase, with one metal ion bound in the M2 position.

E. Tandem mass spectrometry analysis of differentially alkylated tryptic peptides from PHLPP2^{WT} under native and denaturing conditions.

F. Cartesian root-mean-square deviation (RMSD) from the initial equilibrated structure over either all PHLPP2 atoms (red) or distance restrained groups (blue) as a function of time in MD simulations.

G. Validation of the ONIOM method for the experimentally determined C1D3 zinc finger of α-klotho (10) (PDB ID 5W21). Comparison of bond lengths optimized by three quantum mechanical methods, each with different basis sets, to the experimentally determined bond lengths.

H. Comparison of interatomic distances between Zn^{2+} and coordinating atoms in the MS2 binding site (carboxy atoms in aspartic acid or sulfur in cysteine) in the final MD structure and the final QM-optimized structure.

I. B3LYP/6-31+G(3d,p) fully optimized structures of metal binding sites of PHLPP2 comprising ligands from the first and second coordination layer, and enthalpies and Gibbs energies for $[M(H_2O)_6]^{2+}$ + $[PHLPP2\text{-}Zn]^{2-} \rightarrow$ $[PHLPP2\text{-}M]^{2-}$ + $[Zn(H_2O)_6]^{2+}$ (M = Mn or Fe). Solvent accessible metal binding sites characterized with dielectric constant of 29 are considered. Calculation methodology according to (46).

Supplementary Figure 7. Cancer genomics does not support a role for PHLPP1 or PHLPP2 as tumor suppressors.

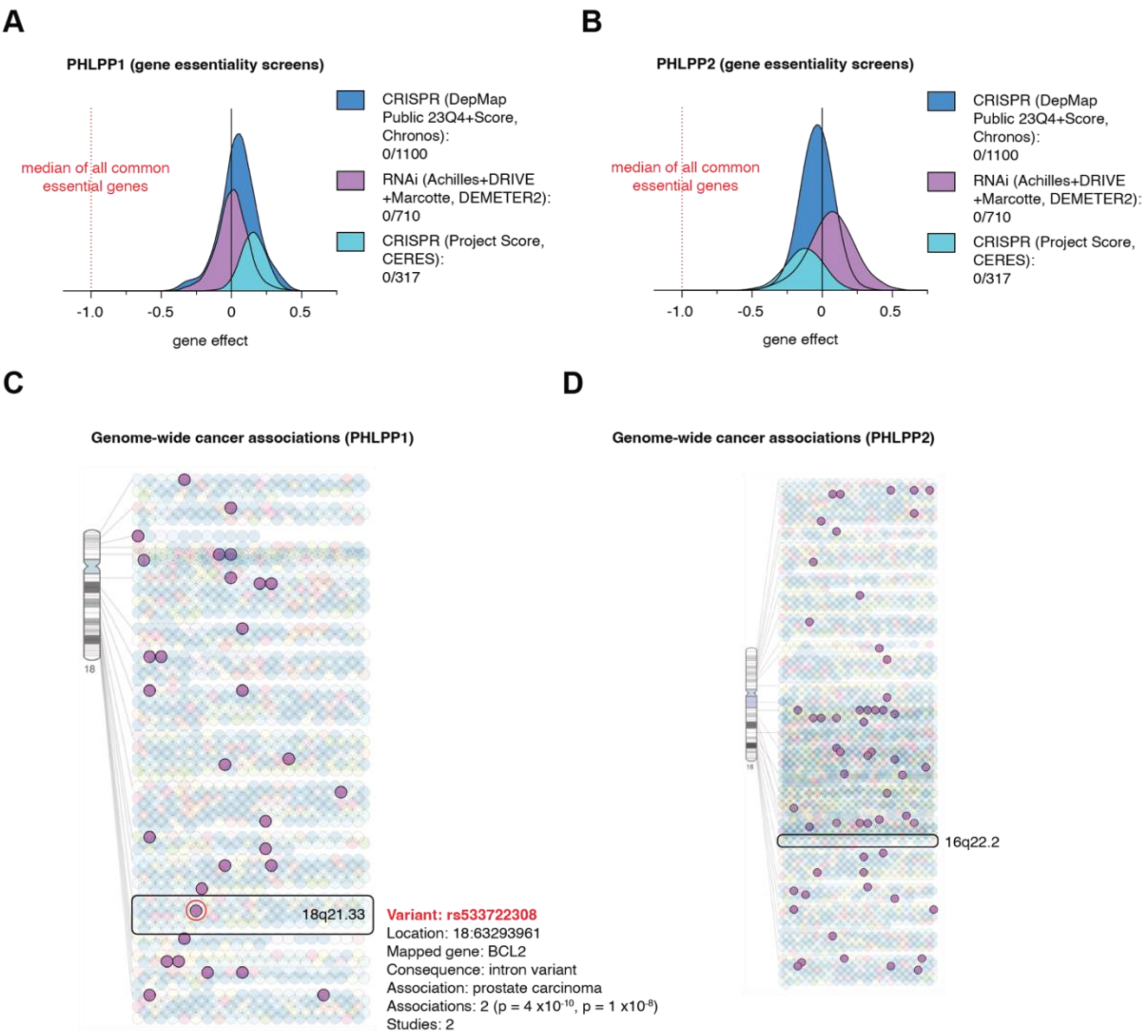

A. PHLPP1 essentiality determined from CRISPR knockout (blue) and RNAi (purple) screens for 1100 and 710 lines respectively. Copy number-corrected CRISPR knockout (CERES) gene effect scores for 317 cancer cell lines (cyan).

B. PHLPP2 essentiality determined from CRISPR knockout (blue) and RNAi (purple) screens for 1100 and 710 lines respectively. Copy number-corrected CRISPR knockout (CERES) gene effect scores for 317 cancer cell lines (cyan).

C. Genome-wide cancer associations in the chromosomal locus 18q21.33 containing the PHLPP1 gene. Red: single cancer-associated variant with a p-value < 5.0 $x10^{-8}$, which maps to an intron of the BCL2 gene.

D. Genome-wide cancer associations in the chromosomal locus 16q22.2 containing the PHLPP2 gene.

Supplementary Figure 8. Cryo-electron microscopy structure of PHLPP2.

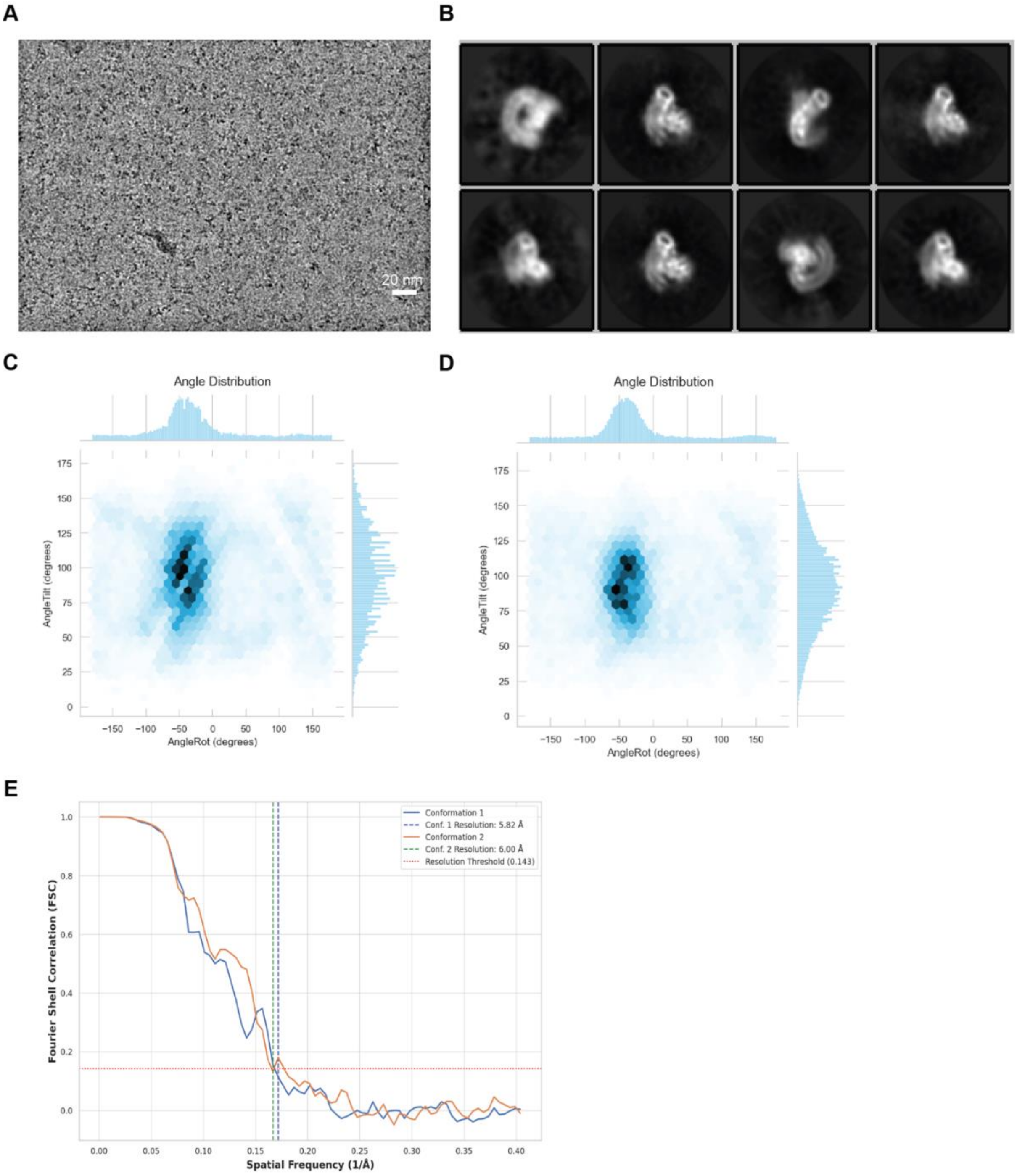

A. Representative micrograph.

B. Selected class averages representing the different viewing directions in the dataset.

C. Angle distribution plot of conformation 1.

D. Angle distribution plot of conformation 2.

E. Fourier shell correlation curves for each conformation.

Supplementary Figure 9. Evolution and diversification of PHLPP genes.

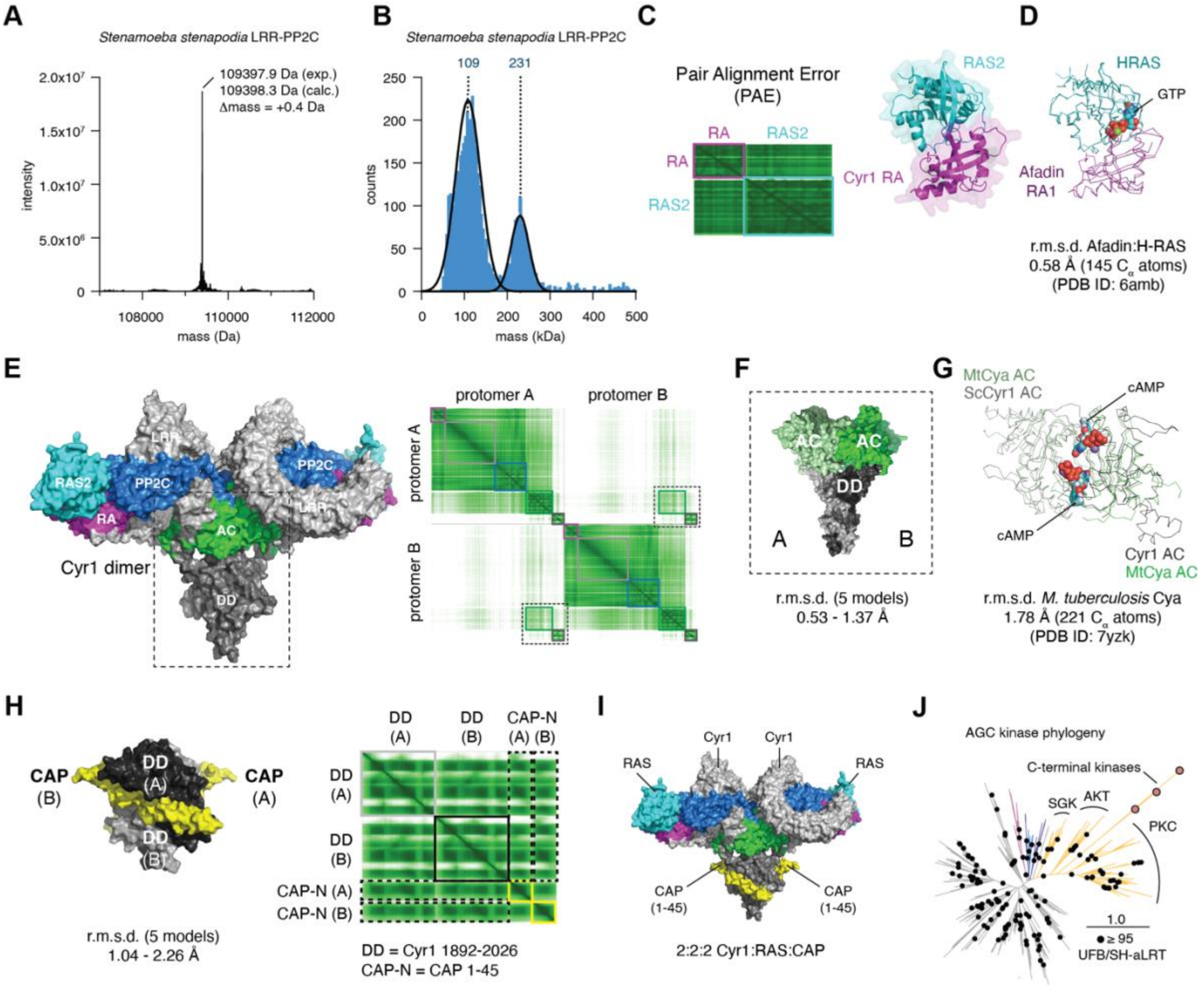

A. Mass spectrum of recombinant *Stenamoeba stenapodia* LRR-PP2C.

B. Mass photometry of recombinant *Stenamoeba stenapodia* LRR-PP2C.

C. Structure prediction and associated PAE plot for the complex between the RA domain of *S. cerevisiae* Cyr1 (magenta) and RAS2 (cyan).

D. Superposition of the predicted Cyr1 RA:RAS2 structure (magenta/cyan) with the experimentally determined structure of the Afadin RA domain with GTP-bound H-RAS (purple/teal) (6amb). R.m.s.d. determined over 145 equivalent C_α atoms.

E. Structure prediction and associated PAE plot for dimeric *S. cerevisiae* Cyr1. Dashed rectangle: dimerization of the AC and CTD domains of Cyr1.

F. Structure prediction and associated PAE plot for dimeric AC-CTD domain of *S. cerevisiae* Cyr1. Agreement of 5 independent models shown by the r.m.s.d. over all C_α atoms.

G. Superposition of the predicted Cyr1 AC-CTD dimer (black/grey) with the experimentally determined structure of the AC domain of *M. tuberculosis* Cya (dark green/light green) (7yzk). R.m.s.d. determined over 221 equivalent C_α atoms.

H. Structure prediction and associated PAE plot for the dimeric CTD domain of *S. cerevisiae* Cyr1 (black/grey) in complex with two molecules of CAP (amino acids 1-45; yellow). Agreement of 5 independent models shown by the r.m.s.d. over all C_α atoms.

I. Structure prediction of the Cyr1:RAS2:CAP heterohexamer (2:2:2).

J. Maximum-likelihood phylogeny of kinase domains. The C-terminal kinase domains of unicellular holozoan Cyr1 cluster with AGC kinases of the Akt and PKC families. The kinase domain phylogeny can be viewed at https://itol.embl.de/shared/9pitR7NesER8.

Supplementary Table 1. Cryo-EM data collection, refinement statistics

	PHLPP conf1 (EMDB-51182)	PHLPP conf2 (EMDB-51183)
**Data collection and processing**		
Magnification	130 k	
Voltage (kV)	300	
Electron exposure (e–/Å^2)	35	
Defocus range (µm)	1.7-2.5	
Pixel size (Å)	0.55 (super resolution)	
Symmetry imposed	C1	
Initial particle images (no.)	958089	
Final particle images (no.)	118375	355036
Map resolution (Å)	6.0	5.8
FSC threshold 0.143		

Supplementary References

1. L. Truebestein, *et al.*, Structure of autoinhibited Akt1 reveals mechanism of PIP3-mediated activation. *Proc. Natl. Acad. Sci. U. S. A.* **118** (2021).

2. E. Y. Chen, *et al.*, Enrichr: interactive and collaborative HTML5 gene list enrichment analysis tool. *BMC Bioinformatics* **14**, 128 (2013).

3. A. Degasperi, *et al.*, Evaluating strategies to normalise biological replicates of Western blot data. *PLoS One* **9**, e87293 (2014).

4. S. Hann, G. Koellensperger, C. Obinger, P. G. Furtmüller, G. Stingeder, SEC-ICP-DRCMS and SEC-ICP-SFMS for determination of metal–sulfur ratios in metalloproteins. *J. Anal. At. Spectrom.* **19**, 74–79 (2004).

5. B. L. Brauer, *et al.*, Affinity-based profiling of endogenous phosphoprotein phosphatases by mass spectrometry. *Nat. Protoc.* **16**, 4919–4943 (2021).

6. M. J. Abraham, *et al.*, GROMACS: High performance molecular simulations through multi-level parallelism from laptops to supercomputers. *SoftwareX* **1–2**, 19–25 (2015).

7. K. Lindorff-Larsen, *et al.*, Improved side-chain torsion potentials for the Amber ff99SB protein force field. *Proteins* **78**, 1950–1958 (2010).

8. S. Piana, A. G. Donchev, P. Robustelli, D. E. Shaw, Water dispersion interactions strongly influence simulated structural properties of disordered protein states. *J. Phys. Chem. B* **119**, 5113–5123 (2015).

9. J. Jumper, *et al.*, Highly accurate protein structure prediction with AlphaFold. *Nature* 1–7 (2021).

10. G. Chen, *et al.*, α-Klotho is a non-enzymatic molecular scaffold for FGF23 hormone signalling. *Nature* **553**, 461–466 (2018).

11. B. Hess, H. Bekker, H. J. C. Berendsen, J. G. E. M. Fraaije, LINCS: A linear constraint solver for molecular simulations. *J. Comput. Chem.* **18**, 1463–1472 (1997).

12. G. Bussi, D. Donadio, M. Parrinello, Canonical sampling through velocity rescaling. *J. Chem. Phys.* **126**, 014101 (2007).

13. M. Parrinello, A. Rahman, Polymorphic transitions in single crystals: A new molecular dynamics method. *J. Appl. Phys.* **52**, 7182–7190 (1981).

14. M. J. Frisch, *et al.*, *Gaussian 16 Rev. C.01* (2016).

15. S. Dapprich, I. Komáromi, K. S. Byun, K. Morokuma, M. J. Frisch, A new ONIOM implementation in Gaussian98. Part I. The calculation of energies, gradients, vibrational frequencies and electric field derivatives1Dedicated to Professor Keiji Morokuma in celebration of his 65th birthday.1. *Journal of Molecular Structure: THEOCHEM* **461–462**, 1–21 (1999).

16. T. Dudev, C. Lim, Factors governing the protonation state of cysteines in proteins: an Ab initio/CDM study. *J. Am. Chem. Soc.* **124**, 6759–6766 (2002).

17. J. Rappsilber, M. Mann, Y. Ishihama, Protocol for micro-purification, enrichment, pre-fractionation and storage of peptides for proteomics using StageTips. *Nat. Protoc.* **2**, 1896–1906 (2007).

18. A. T. Kong, F. V. Leprevost, D. M. Avtonomov, D. Mellacheruvu, A. I. Nesvizhskii, MSFragger: ultrafast and comprehensive peptide identification in mass spectrometry-based proteomics. *Nat. Methods* **14**, 513–520 (2017).

19. D. M. Hollenstein, *et al.*, Chemical Acetylation of Ligands and Two-Step Digestion Protocol for Reducing Codigestion in Affinity Purification-Mass Spectrometry. *J. Proteome Res.* **22**, 3383–3391 (2023).

20. Y. Perez-Riverol, *et al.*, The PRIDE database and related tools and resources in 2019: improving support for quantification data. *Nucleic Acids Res.* **47**, D442–D450 (2019).

21. A. Punjani, J. L. Rubinstein, D. J. Fleet, M. A. Brubaker, cryoSPARC: algorithms for rapid unsupervised cryo-EM structure determination. *Nat. Methods* **14**, 290–296 (2017).

22. D. Kimanius, L. Dong, G. Sharov, T. Nakane, S. H. W. Scheres, New tools for automated cryo-EM single-particle analysis in RELION-4.0. *Biochem. J* **478**, 4169–4185 (2021).

23. D. Kimanius, *et al.*, Data-driven regularisation lowers the size barrier of cryo-EM structure determination. *bioRxiv* 2023.10.23.563586 (2023).

24. T. D. Goddard, *et al.*, UCSF ChimeraX: Meeting modern challenges in visualization and analysis. *Protein Sci.* **27**, 14–25 (2018).

25. R. T. Kidmose, *et al.*, Namdinator - automatic molecular dynamics flexible fitting of structural models into cryo-EM and crystallography experimental maps. *IUCrJ* **6**, 526–531 (2019).

26. UniProt Consortium, UniProt: a worldwide hub of protein knowledge. *Nucleic Acids Res.* **47**, D506–D515 (2019).

27. F. A. Simão, R. M. Waterhouse, P. Ioannidis, E. V. Kriventseva, E. M. Zdobnov, BUSCO: assessing genome assembly and annotation completeness with single-copy orthologs. *Bioinformatics* **31**, 3210–3212 (2015).

28. B. Buchfink, K. Reuter, H.-G. Drost, Sensitive protein alignments at tree-of-life scale using DIAMOND. *Nat. Methods* **18**, 366–368 (2021).

29. S. Capella-Gutiérrez, J. M. Silla-Martínez, T. Gabaldón, trimAl: a tool for automated alignment trimming in large-scale phylogenetic analyses. *Bioinformatics* **25**, 1972–1973 (2009).

30. S. Guindon, *et al.*, New algorithms and methods to estimate maximum-likelihood phylogenies: assessing the performance of PhyML 3.0. *Syst. Biol.* **59**, 307–321 (2010).

31. K. Katoh, D. M. Standley, MAFFT Multiple Sequence Alignment Software Version 7: Improvements in Performance and Usability. *Mol. Biol. Evol.* **30**, 772–780 (2013).

32. L. T. Nguyen, H. A. Schmidt, A. Von Haeseler, B. Q. Minh, IQ-TREE: A fast and effective stochastic algorithm for estimating maximum-likelihood phylogenies. *Mol. Biol. Evol.* **32**, 268–274 (2015).

33. D. T. Hoang, O. Chernomor, A. von Haeseler, B. Q. Minh, L. S. Vinh, UFBoot2: Improving the Ultrafast Bootstrap Approximation. *Mol. Biol. Evol.* **35**, 518–522 (2018).

34. D. J. Richter, *et al.*, EukProt: A database of genome-scale predicted proteins across the diversity of eukaryotes. *Peer Community Journal* **2** (2022).

35. S. Kalyaanamoorthy, B. Q. Minh, T. K. F. Wong, A. Von Haeseler, L. S. Jermiin, ModelFinder: Fast model selection for accurate phylogenetic estimates. *Nat. Methods* **14**, 587–589 (2017).

36. F. Burki, A. J. Roger, M. W. Brown, A. G. B. Simpson, The New Tree of Eukaryotes. *Trends Ecol. Evol.* **35**, 43–55 (2020).

37. H. Shimodaira, An approximately unbiased test of phylogenetic tree selection. *Syst. Biol.* **51**, 492–508 (2002).

38. I. Letunic, P. Bork, Interactive Tree of Life (iTOL) v6: recent updates to the phylogenetic tree display and annotation tool. *Nucleic Acids Res.* (2024). https://doi.org/10.1093/nar/gkae268.

39. J. Mistry, R. D. Finn, S. R. Eddy, A. Bateman, M. Punta, Challenges in homology search: HMMER3 and convergent evolution of coiled-coil regions. *Nucleic Acids Res.* **41**, e121 (2013).

40. J. Mistry, *et al.*, Pfam: The protein families database in 2021. *Nucleic Acids Res.* **49**, D412–D419 (2021).

41. T. J. Wheeler, J. Clements, R. D. Finn, Skylign: a tool for creating informative, interactive logos representing sequence alignments and profile hidden Markov models. *BMC Bioinformatics* **15**, 7 (2014).

42. B. Boeckmann, *et al.*, The SWISS-PROT protein knowledgebase and its supplement TrEMBL in 2003. *Nucleic Acids Res.* **31**, 365–370 (2003).

43. G. S. C. Slater, E. Birney, Automated generation of heuristics for biological sequence comparison. *BMC Bioinformatics* **6**, 31 (2005).

44. N. A. T. Irwin, *et al.*, The function and evolution of motile DNA replication systems in ciliates. *Curr. Biol.* **31**, 66-76.e6 (2021).

45. T. Gao, F. Furnari, A. C. Newton, PHLPP: a phosphatase that directly dephosphorylates Akt, promotes apoptosis, and suppresses tumor growth. *Mol. Cell* **18**, 13–24 (2005).

46. T. Dudev, K. Mazmanian, C. Lim, Competition between Li+ and Na+ in sodium transporters and receptors: Which Na+-Binding sites are “therapeutic” Li+ targets? *Chem. Sci.* **9**, 4093–4103 (2018).
